# Intracranial tuberculomas or neurocysticercosis: differentiated by cervical lymph node pathology

**DOI:** 10.1186/s41983-022-00554-x

**Published:** 2022-10-10

**Authors:** Sevda Diker, Derlen Özgeç Ruso, Nesil Bayraktar, Uğurcan Balyemez

**Affiliations:** 1 Faculty of Medicine, Department of Neurology, Cyprus International University, Nicosia, Cyprus; 2 Department of Chest Diseases, Dr. Burhan Nalbantoğlu State Hospital, Nicosia, Cyprus; 3 Department of Infectious Diseases and Clinical Microbiology, Dr. Burhan Nalbantoğlu State Hospital, Nicosia, Cyprus; 4 Faculty of Medicine, Department of Radiology, Near East University, Nicosia, Cyprus

**Keywords:** Tuberculoma, Seizure, Neurocysticercosis, Magnetic resonance imaging

## Abstract

**Background:**

Diagnosis of tuberculomas can be difficult in the absence of pulmonary involvement due to numerable mimics.

**Case report:**

We report an immunocompetent 20-year-old female patient, who was admitted with new-onset generalized seizure. Cranial magnetic resonance imaging (MRI) revealed multiple ring-enhancing lesions. There was no reported systemic symptom such as weight loss, fever or night sweating. Polymerase chain reaction for SARS-COV-2 was negative. Computed tomography of thorax was normal. With an initial diagnosis of neurocysticercosis, she was treated with albendazole for one month. Follow-up cranial MRI showed no improvement. On follow-up visit, an enlarged cervical lymph node was recognized. Biopsy of the lymph node led to the diagnosis of tuberculosis. Two months after the onset of anti-tuberculosis therapy, follow-up cranial MRI showed near-complete resolution.

**Conclusion:**

Investigation of any involvement of disease other than the central nervous system can enable accurate and timely diagnosis of tuberculomas in the absence of pulmonary involvement.

## Background

Tuberculosis (TB) is a devastating communicable disease caused by *Mycobacterium tuberculosis.* The highest rates of TB are observed in sub-Saharan Africa, India, and the islands of Southeast Asia and Micronesia.

Of the extrapulmonary manifestations of tuberculosis, central nervous system (CNS) involvement, seen in approximately one percent of all TB cases, is a rare but highly devastating manifestation with a high rate of mortality, as well as neurologic sequelae despite treatment. The usual presentation of CNS TB is tuberculous meningoencephalitis (TBM). Tuberculoma is the most common form of intracranial parenchymal TB; however, it is seen rarely [1, 2].

The radiographic characteristic of tuberculomas is ring-enhancing lesions, which often mimic those of other infectious and non-infectious conditions. Neurocysticercosis (NCC) is the most dilemmatic differential with similar epidemiologic, clinical, and radiographic features.

Herein, we report an immunocompetent female patient who presented with seizure and an MRI revealing dominantly ring-enhancing lesions. Although she was previously misdiagnosed as having NCC, a comprehensive systemic evaluation revealed a final diagnosis of intracranial tuberculomas.

## Case presentation

**Fig. 1:**
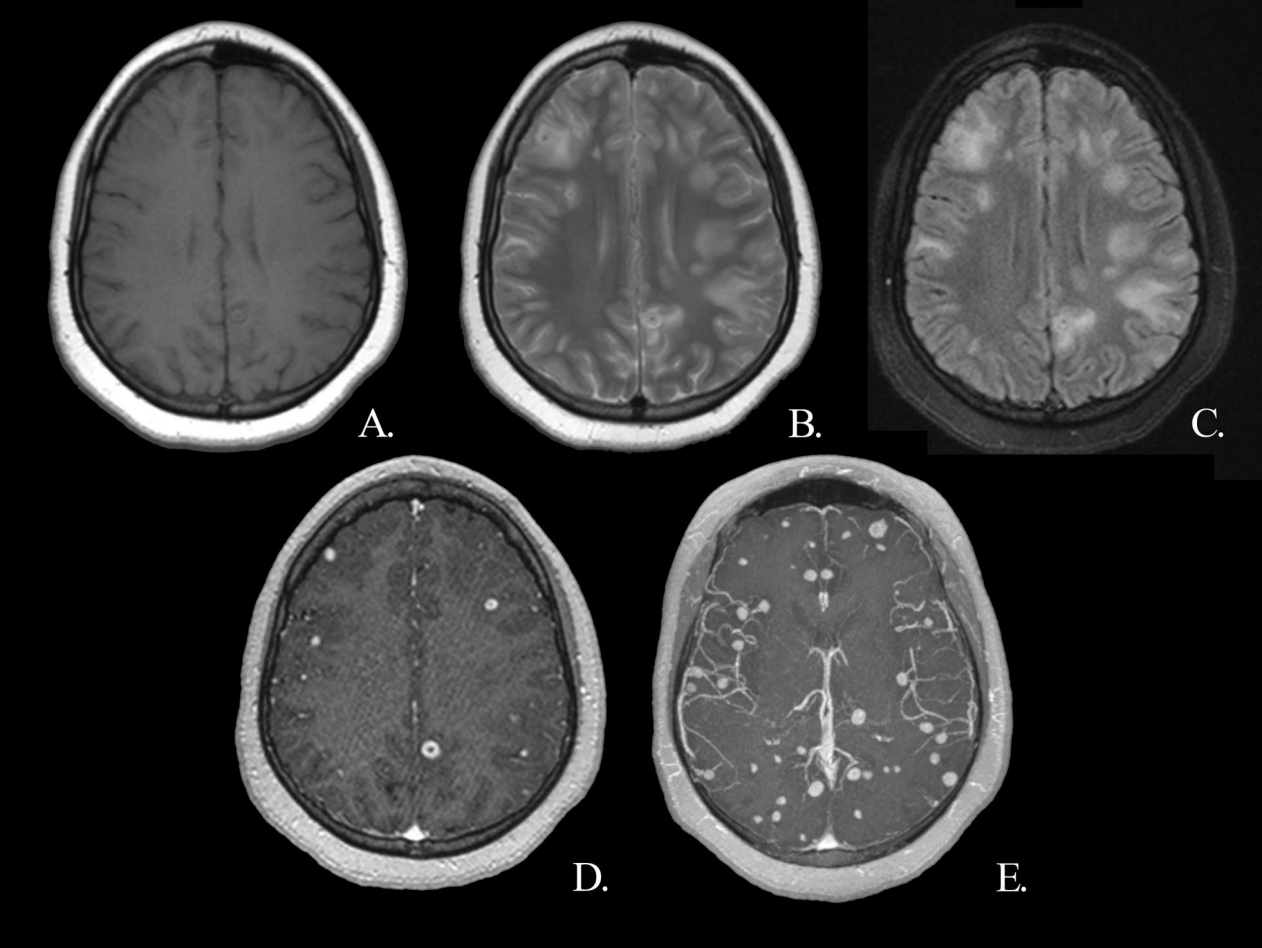
Patient's first MRI on admission. Multiple hypointense, nodular lesions ranging in size from 5 to 9 mm were observed on T1W (**A**), T2W (**B**), and FLAIR **C** images, showing infratentorial and supratentorial distribution with partial preservation of the central parts. The lesions did not show diffusion restriction (not shown) and there was surrounding vasogenic edema, which was observed as a patchy signal increase on T2 and FLAIR images (**B**, **C**). There was no signal loss in the suscebtibility-weighted images (not shown). In contrast-enhanced images, ring-like enhancement was observed mostly, and homogeneous nodular enhancement was observed in a few smaller lesions (**D**). Note the high number and watershed localization of the lesions on post-contrast T1W axial MIP images (**E**). *FLAIR* fluid-attenuated inversion recovery, *MIP* maximum intensity projection, *T1W* T1 weighted, *T2W* T2 weighted

A previously healthy twenty year-old female patient was admitted to outpatient clinic after she had experienced a generalized tonic–clonic seizure while she was sleep-deprived. Her neurologic examination and vital signs were normal. Laboratory tests were within normal limits except C-reactive protein which was elevated at 45.8 (normal range: 0–6) mg/L. She reported no recent fever, signs of upper respiratory, urinary or gastrointestinal infection. She only reported hip pain. X-rays of the thorax, hip, and lumbar region were reported as normal. Electrencephalography (EEG) revealed generalized epileptiform pathology. Levetiracetam 500 mg tablets twice per day was started. On MRI, multiple ring-enhancing lesions were observed (Fig. 1).

Considering an infectious etiology, the patient was referred to the infectious diseases department where albendazole treatment was initiated with a probable diagnosis of NCC. Although the diagnosis of NCC typically requires both CNS imaging and serological testing including enzyme-linked immunoelectrotransfer blot (EITB), and commercial enzyme-linked immunoassays, serological tests were not performed because they were not readily available. Due to the present symptoms and radiological findings compatible with NCC, urgent treatment was started.

In the fourth week of albendazole treatment, the patient was admitted to our hospital with new-onset fever while receiving antiparasitic treatment. She denied having cough, loss of appetite, weight loss or night sweats. Her neurologic examination was normal without any sign of increased intracranial pressure or meningeal irritation. Her seizures were under control with levetirasetam. A polymerase chain reaction (PCR) test for coronavirus was negative. Thoracic scans were normal. Repeated cranial MRI showed no improvement compared with the initial imaging performed one month ago.

With a suspicion of *Mycobacterium tuberculosis* infection, QuantiFERON-TB Gold test was performed and resulted positive indicating previous TB infection. MRI of the hip revealed active severe sacroiliitis on the right side. An enlarged lymph node was newly detected in the anterior cervical region. Biopsy of the lymph node showed granulomatous lymphadenitis including necrotizing Langhans-type giant cells, which was compatible with a diagnosis of TB. Anti-HIV (human immunodeficiency virus) antibody testing was negative and serum immunoglobulin levels (IgG,A,M) were within normal limits. Antituberculosis treatment with isoniazid—plus pyridoxine, rifampicin, ethambutol, and pyrazinamide was started.

**Fig. 2:**
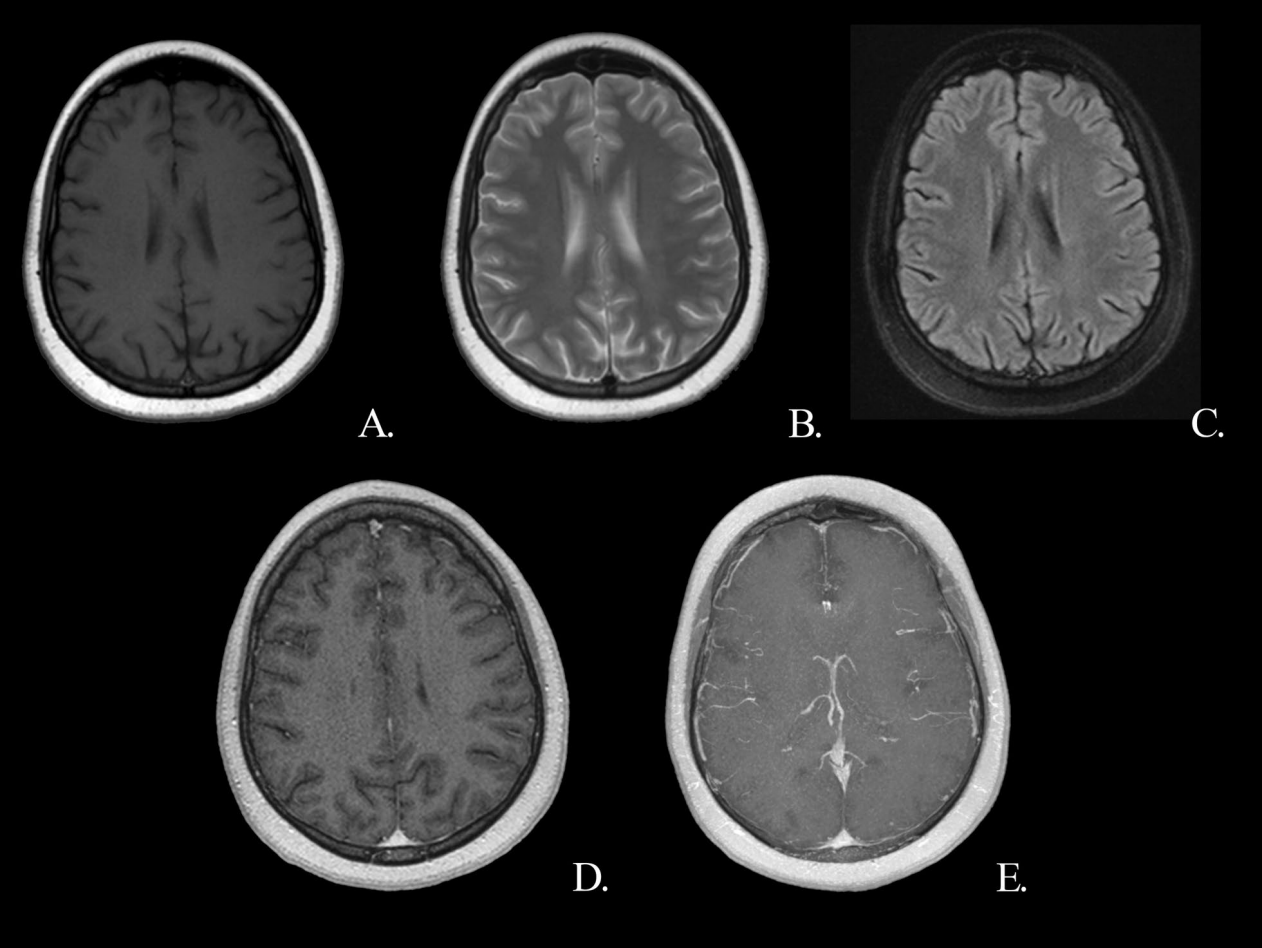
Control MRI after 2 months of anti-tuberculosis theraphy demonstrates resolution of the lesions. Precontrast T1W axial (**A**), T2W axial (**B**), FLAIR axial (**C**), post-contrast T1W axial (**D**), post-contrast T1W axial MIP images (**E**). *FLAIR* fluid-attenuated inversion recovery, *MIP* maximum intensity projection, *T1W* T1 weighted, *T2W* T2 weighted

Two months after the onset of anti-tuberculosis therapy, follow-up cranial MRI showed near-complete resolution of the tuberculomas in the brain (Fig. 2). We planned to continue four drugs for a total of six months; after six months, isoniazid and rifampicin for a further 12 months.

## Conclusion

Our patient had several tuberculomas without meningeal inflammation and presented with a seizure, which is the most common symptom of tuberculomas. Unlike TB meningitis, tuberculomas are often clinically silent and the patients often look clinically well. Patients can present with seizure, headache, signs of intracranial hypertension, or focal neurologic symptoms based on the topographic location of the lesions [3].

The radiographic characteristics of tuberculomas often mimic those of other infectious and non-infectious conditions, and the clinical manifestations are diverse and nonspecific. Important differential diagnoses include neurocysticercosis, metastasis, CNS lymphoma (in immunocompromised patients), toxoplasmosis, tumours (such as glioblastoma) and pyogenic abscess [1]. In clinical practice, although most of these diagnoses can be distinguished using MRI features, it would be not easy to decide between two close differentials, NCC and tuberculoma.

Tuberculoma and NCC share similar not only clinical and radiographic, but also epidemiologic features. Our patient, until the last 3 months, had been living in the Democratic Republic of Congo where tuberculosis and neurocycticercosis are highly relevant. Both tuberculoma and NCC can present with seizures and multiple nodular or ring-enhancing lesions in the brain without pulmonary involvement.

MRI in tuberculomas shows conglomerate ring-enhancing lesions, which are located in the grey-white matter junction, mostly located in the frontal and parietal regions. They have a larger extent of perilesional edema. Unlike NCC, they are hypo/isointense lesions with a hypointense rim on T2-weighted images, iso/hypointense on T1-weighted images, with no/incomplete suppression on fluid attenuated recovery (FLAIR) images [1].

In NCC, cranial MRI reveals single or multiple lesions either cystic with scolex or in different stages of evolution [4]. Detection of eccentric T2 hypointense scolex is an absolute diagnostic criterion for NCC; however, it is not usually seen. Although cysts are T2 hyperintense unless calcified and show complete suppression on FLAIR [1] unlike tuberculomas, degenerating cysts appear as contrast-enhancing rings or nodules surrounded by mild edema mimicking tuberculoma. Besides common radiological features, biochemical and cytologic examinations of cerebrospinal fluid (CSF), and serum and CSF antibody testing can be normal for both in the early disease stages [5].

Although radiological findings depend on the location and stage of the disease, computed tomography (CT) is superior to MRI in identifying calcified granulomas and help in differential diagnosis [6]. The utility of magnetic resonance spectroscopy (MRS) has also been demonstrated. MRS showed a grossly diminished N-acetyl aspartate peak and a specific lipid peak in tuberculoma cases, whereas in the normal brain, MRS showed that the N-acetyl aspartate peak was the highest, and there was no lipid or lactate peak. In contrast, in cases of NCC, moderately diminished N-acetyl aspartate and the presence of a lactate peak but the absence of a lipid peak were observed. Therefore, a high lipid peak on MRS is highly specific tuberculoma in the context of ring-enhancing lesions [7]. It has also been suggested that tuberculomas can be distinguished from NCC based on a choline/creatine ratio of > 1 [8].

A definitive diagnosis of tuberculoma is established via needle biopsy of the CNS lesion for histopathology and acid-fast bacilli (AFB) stain and culture. However, surgical intervention is often impractical. The tuberculin skin test and interferon gamma releasing assays like ‘QuantiFERON-TB gold’ indicate previous TB infection and neither is sufficiently sensitive nor specific to diagnose active disease. AFB staining, TB-PCR, and culture of CSF for TBC can be negative especially in the early stages [5]. Moreover, lumbar puncture can be avoided because of the concern for elevated intracranial pressure. This very challenge underscores the importance of detecting adjunctive, extracranial evidence of active TB which support the diagnosis of intracranial tuberculomas.

In summary, the diagnosis of tuberculomas in the brain may be challenging because of various mimics. Careful evaluation of a patient’s history, demographic features, and interpretation of MRI findings are crucial for differential diagnosis and for directing the clinician to appropriate diagnostic tests. As highlighted by our case, investigation of any involvement of disease other than CNS can enable accurate and timely diagnosis of tuberculomas in the absence of pulmonary involvement.

## Data Availability

The data used and/or analysed during the current case report are available from the corresponding author on reasonable request.

## Abbreviations

AFB: Acid-fast bacilli
CNS: Central nervous system
CSF: Cerebrospinal fluid
EEG: Electrencephalography
FLAIR: Fluid-attenuated inversion recovery
MRI: Magnetic resonance imaging
NCC: Neurocysticercosis
PCR: Polymerase chain reaction
TB: Tuberculosis
TBM: Tuberculous meningoencephalitis
